# A randomised trial examining inflammatory signaling in acutely induced hyperinsulinemia and hyperlipidemia in normal weight women-the reprometabolic syndrome

**DOI:** 10.1371/journal.pone.0247638

**Published:** 2021-03-25

**Authors:** Andrew Tannous, Andrew P. Bradford, Katherine Kuhn, Angela Fought, Irene Schauer, Nanette Santoro

**Affiliations:** 1 Department of Obstetrics & Gynecology, Division of Endocrinology Metabolism and Diabetes, University of Colorado School of Medicine, Aurora, CO, United States of America; 2 Department of Medicine, Division of Endocrinology Metabolism and Diabetes, University of Colorado School of Medicine, Aurora, CO, United States of America; 3 Endocrinology Section, Rocky Mountain Regional Veterans Affairs Medical Center, Aurora, CO, United States of America; Weill Cornell Medical College Qatar, QATAR

## Abstract

**Context:**

Obesity, is a state of chronic inflammation, characterized by elevated lipids, insulin resistance and relative hypogonadotropic hypogonadism. We have defined the accompanying decreased Luteinizing Hormone (LH), Follicle-Stimulating Hormone (FSH), ovarian steroids and reduced pituitary response to Gonadotropin-releasing Hormone (GnRH) as Reprometabolic syndrome, a phenotype that can be induced in healthy normal weight women (NWW) by acute infusion of free fatty acids and insulin.

**Objective:**

To identify potential mediators of insulin and lipid-related reproductive endocrine dysfunction.

**Design, setting, participants:**

Secondary analysis of crossover study of eumenorrheic reproductive aged women of normal Body Mass Index (BMI) (<25 kg/m^2^) at an academic medical center.

**Intervention:**

Participants underwent 6-hour infusions of either saline/heparin or insulin plus fatty acids (Intralipid plus heparin), in the early follicular phase of sequential menstrual cycles, in random order. Euglycemia was maintained by glucose infusion. Frequent blood samples were obtained.

**Main outcome measures:**

Pooled serum from each woman was analyzed for cytokines, interleukins, chemokines, adipokines, Fibroblast Growth Factor-21 (FGF-21) and markers of endoplasmic reticulum (ER) stress (CHOP and GRP78). Wilcoxon signed-rank tests were used to compare results across experimental conditions.

**Results:**

Except for Macrophage Inflammatory Protein-1β (MIP-1β), no significant differences were observed in serum levels of any of the inflammatory signaling or ER stress markers tested.

**Conclusion:**

Acute infusion of lipid and insulin, to mimic the metabolic syndrome of obesity, was not associated with an increase in inflammatory markers. These results imply that the endocrine disruption and adverse reproductive outcomes of obesity are not a consequence of the ambient inflammatory environment but may be mediated by direct lipotoxic effects on the hypothalamic-pituitary-ovarian (HPO) axis.

## Introduction

Obesity leads to menstrual abnormalities, infertility, miscarriage, and complications in assisted reproduction [1]. Women with obesity are at high risk for insulin resistance and hyperinsulinemia, altered gonadotrophin secretion, decreased sex hormone binding globulin, and altered neuroregulation of the hypothalamic-pituitary-gonadal axis [2]. Although obesity has been implicated in reproductive dysfunction, the pathophysiology is not fully understood. We have previously shown that there is a decrease in amplitude of pulsatile LH and reduced FSH secretion in women with obesity relative to NWW [3]. The defects in gonadotropin secretion are due, at least in part, to blunted pituitary response to exogenous GnRH [4], and appear to improve with surgically induced weight loss [5]. Taken together, these data suggest that obesity induces a functional impairment of the hypothalamic-pituitary-ovarian axis at the level of the pituitary gland, leading to an observed impact on FSH and LH secretion and downstream adverse effects on reproductive hormone secretion. The isolated dysfunction of the pituitary gland, which is exposed to the peripheral circulation, in turn implies that circulating factors present in the bloodstream are mediating the previously observed blunting of LH and FSH responses to GnRH in obesity.

We have previously demonstrated that the combination of infused free fatty acids and insulin, administered via a hyperinsulinemic, euglycemic clamp, acutely reduces serum LH and FSH in healthy, non-obese men and women [6]. These conditions of hyperinsulinemia and free fatty acidemia recapitulate elements of the metabolic syndrome, including development of insulin resistance [6]. Given the concomitant alterations in reproductive hormone patterns we have adopted the term ‘reprometabolic syndrome’ to describe the metabolic dysfunction and relative hypogonadotrophic hypogonadism characteristic of women with obesity. Herein, we sought to determine the underlying cause for the rapid (within 6 hours) induction of reprometabolic syndrome in normal weight women. Because obesity is a state of chronic low-grade inflammation, we hypothesized that inflammatory signaling to the pituitary may impair gonadotropin synthesis and secretion and explain the previously demonstrated link between obesity and infertility [7, 8]. We have previously shown that low dose transdermal estrogen pre-treatment selectively reduced inflammatory cytokines in women with obesity accompanied by an increase in LH pulse amplitude and GnRH stimulated FSH [4].

To identify potential mediators of insulin and free fatty acid-related reproductive endocrine dysfunction, we investigated cytokine profiles in NWW during both a saline/heparin (control) infusion or a lipid/heparin infusion with a concurrent hyperinsulinemic, euglycemic clamp (HEC). Additionally, given the evidence of the effects of dietary fatty acids on the extra-cellular expression of ER stress markers [9] and the documented effects on the HPO axis [10], we measured a key protein, FGF-21, that has been implicated in the regulation of insulin sensitivity and adiposity [11].

## Methods

### Participants

This study was performed as a secondary analysis of an ongoing study. Parent study recruitment consisted of normal weight (BMI 18–24.9 kg/m^2^) women in good health, aged 18–38 years old, who reported no more than 4 hours of vigorous exercise per week. All women were premenopausal, with a history of regular menstrual cycles, and underwent study visits during the early follicular phase of the menstrual cycle (Days 2–5 after the onset of menses). Participants had normal serum prolactin and thyroid stimulating hormone levels. Exclusionary comorbid conditions included hypertension, pregnancy, overt diabetes, screening triglycerides >250mg/dl, liver or kidney disease, pulmonary disease, chronic inflammatory conditions, coagulopathy, anemia, abnormal cardiac function or evidence of ischemic heart disease. Additional exclusions included: use of medications known to impact insulin production or sensitivity, the presence of soy or egg allergies (due to possible reaction to the lipid infusate), tobacco use, and use of any medications or supplements that would impact reproductive hormones, including systemic hormonal contraception. The study was approved by the University of Colorado Multiple Institutional Review Board (COMIRB) and all participants provided informed consent for the study procedures as outlined. The study is registered at clinicaltrials.gov (NCT02653092).

### Study design

**Fig 1:**
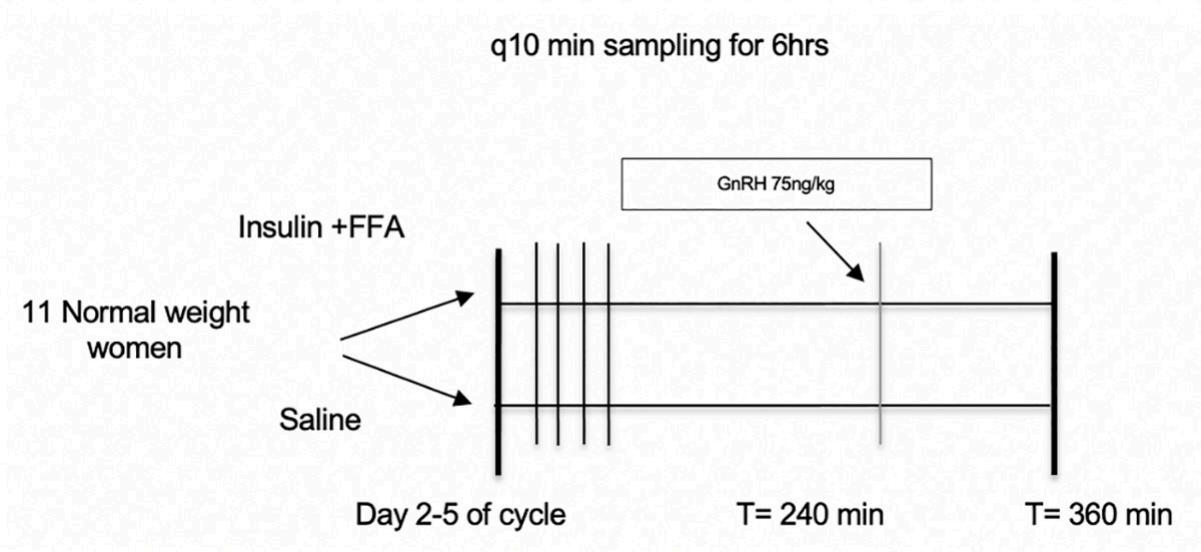
Depiction of each study arm and timeline. Crossover study of 11 NWW. Bolus of GnRH administered at 240 min in both visits, done to measure pulsatility as part of related but separate study.

Eleven regularly cycling, non-diabetic NWW who met inclusion criteria were studied (see Participants section) from February 2016 to March 2020. Each participant underwent two treatments; in random order: a control (saline/heparin infusion) or a hyperinsulinemic euglycemic clamp with intralipid/heparin infusion (insulin:40mU/m^2^/min; lipid [20% lipid emulsion, 45cc/hr]), during a 6-hour frequent blood sampling (q10 min) study, performed during the early follicular phase of separate menstrual cycles as described previously (Santoro, et al Endocrine Society 2018, Abstract 3399) (Fig 1). Heparin (0.4U/kg/min) was administered with lipid infusion to specifically increase free fatty acid levels through the release of lipoprotein lipase into the circulation. Heparin was therefore also included with the saline infusion to control for potential confounding effects of heparin. The treatment order was determined randomly using GraphPad QuickCals online calculator by the study biostatistician and given to the coordinator prior to the first study visit. The participants were blinded to which treatment would be performed first. Study staff assigned the treatment once the participant qualified; 5 participants underwent the saline infusion first. After each blood draw, samples were immediately centrifuged at 3000 rpm for 10 min. Serum was aliquoted and stored in non-absorbent, polypropylene tubes at -80°C until required. For the purpose of this study, we combined aliquots of each woman’s q10’ blood samples between time t = 180 and t = 230, after steady-state conditions were achieved, to perform all measurements. The sample size of 11 was determined based on previous studies [6].

### Assays

A complete summary of measured cytokines, growth factors, ER stress markers and their respective limits of detection are provided in Table 1. Eotaxin, Eotaxin-3, granulocyte-macrophage colony stimulating factor (GM-CSF), interferon-gamma (IFN-γ), interleukins IL-1α, IL-1β, IL-2, IL-4, IL-5, IL-6, IL-7, IL-8, IL-10, IL-12/IL-23p40, IL-12p70, IL-13, IL-15, IL-16, IL-17A, IP-10, monocyte chemotactic proteins (MCP-1, MCP-4), macrophage-derived chemokine (MDC), macrophage inflammatory proteins (MIP-1α, MIP-1β), thymus and activation regulated chemokine (TARC), tumor necrosis factor (TNF-α, TNF-β), and vascular endothelial growth factor-A (VEGF-A) were measured using the V-Plex human cytokine 30-plex immunoassay (Meso Scale Discovery, Rockville, MD) Inter-assay and intra-assay coefficients of variation (CV) for each analyte are <7% and <15%, respectively.

**Table 1:** Serum analytes with limits of detection.

Cytokines, Growth factors, ER-stress markers				
**IFN-γ**: 0.02	**IL-10**: 0.03	**IL-5**: 0.22	**TNF-β**: 0.05	**IP-10**: 0.37
**IL-1β**: 0.04	**IL-12p70**: 0.11	**IL-7**: 0.16	**VEGF**: 1.12	**MIP-1α**: 3.02
**IL-2**: 0.09	**IL-13**: 0.24	**IL-12/IL-23p40**: 0.39	**Eotaxin**: 3.26	**IL-8(HA)**: 95.6
**IL-4**: 0.02	**TNF-α**: 0.04	**IL-15**: 0.17	**MIP-1β**: 0.37	**MCP-1**: 0.09
**IL-6**: 0.06	**GM-CSF**: 0.14	**IL-16**: 2.83	**Eotaxin-3**: 1.77	**MDC**: 1.22
**IL-8**: 0.04	**IL-1α**: 0.09	**IL-17A**: 0.74	**TARC**: 0.22	**MCP-4**: 1.69
**FGF-21**: 0.019*	**CHOP**: 0.124*	**GRP78**: 8.4*		

Values are in pg/ml or *ng/ml as indicated.

FGF-21 was measured by Enzyme-Linked Immunosorbent Assay (ELISA; Abcam, Cambridge, MA). Inter-assay and intra-assay CV are 7.2% and 4.7%, respectively. GRP78 and CHOP were measured as described [12]. GRP78/BiP was measured by ELISA (Enzo, Farmingdale, NY). CHOP ELISA was obtained from LSBio, (Seattle, WA). Manufacturer’s reported inter- and intra-assay CVs for CHOP are <12.0% and <10%. All samples and external standards were run in duplicate. C-reactive protein (CRP) was measured by University Hospital Core laboratory.

### Statistical analysis

Wilcoxon signed-rank tests [13] were used to determine differences between cytokines in the lipid/insulin and saline infusions, allowing each participant to serve as her own control and to minimize between-woman variation. We report p values for each comparison and the level of significance was set at p < 0.05 for all analyses with no adjustments for multiple comparisons.

## Results

**Fig 2:**
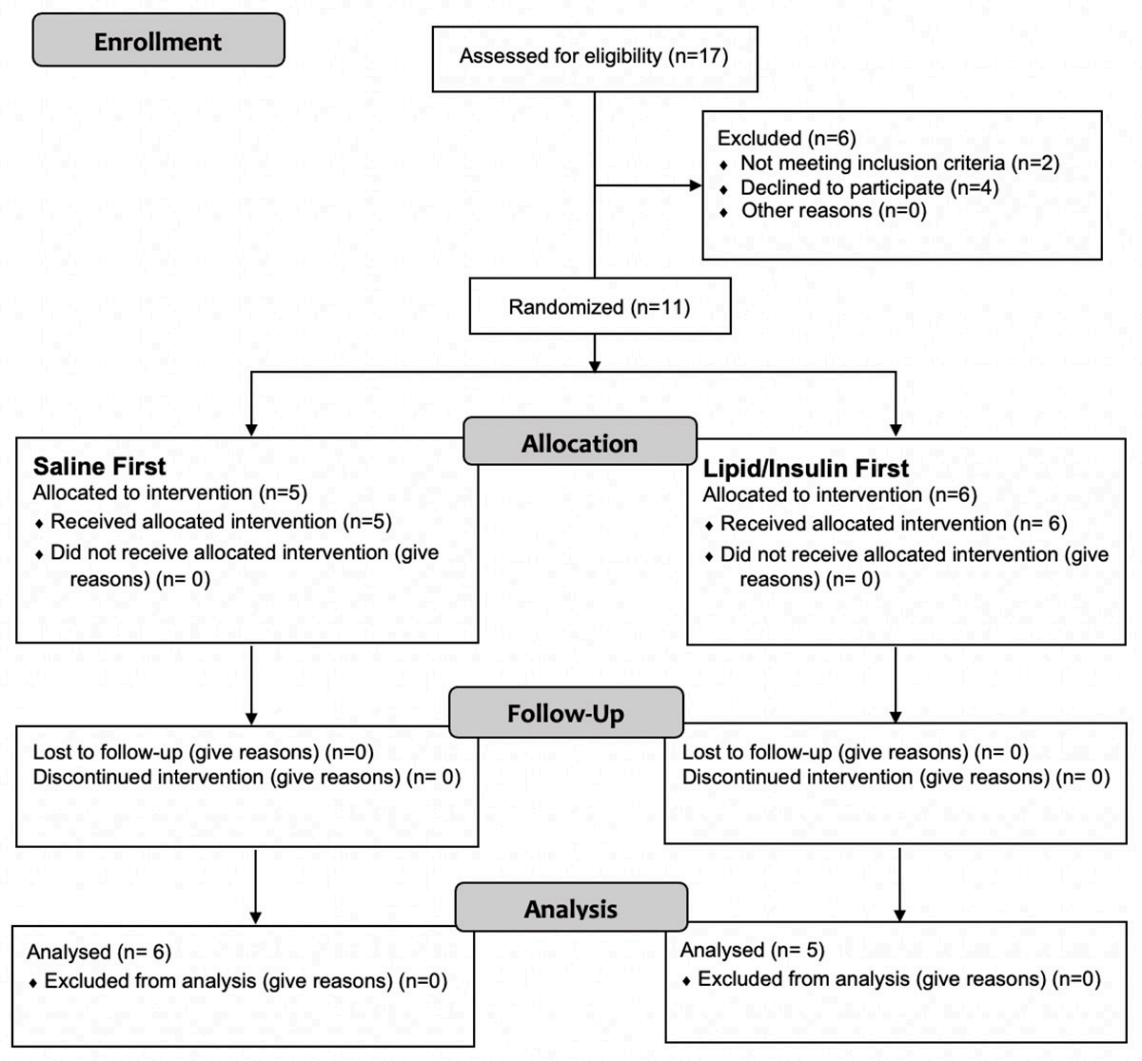
Flow diagram showing process for participants screening, enrollment and randomization for study visit infusions. The total participants analyzed was 11.

Eleven NWW were included in the study and analyzed as depicted in Fig 2. Baseline demographics and clinical characteristics of the participants are depicted in Table 2.

**Table 2:** Baseline characteristics of participants.

Parameter	Mean ± SD
Total Enrollment	11
Age (years)	26.33 ± 4.87
BMI (kg/m^2^)	22.22 ± 1.32
Weight (kg)	60.61 ± 3.75
Height (cm)	165.4 ± 4.8
Waist circumference	69.4 ± 15.3
Waist-Hip Ratio	0.83 ± 0.09
TSH (mIU/ml)	1.43 ± 0.46
Prolactin (ng/ml)	9.68 ± 3.99
AMH (ng/ml)	4.59 ± 3.08
Cycle Length (days)	27.5 ± 1.4
Fasting glucose (mg/dl)	80.4 ± 4.9
Hemoglobin A1c (%)	5.1 ± 0.4
Systolic BP (mmHg)	110 ± 8
Diastolic BP (mmHg)	65 ± 9

**Fig 3:**
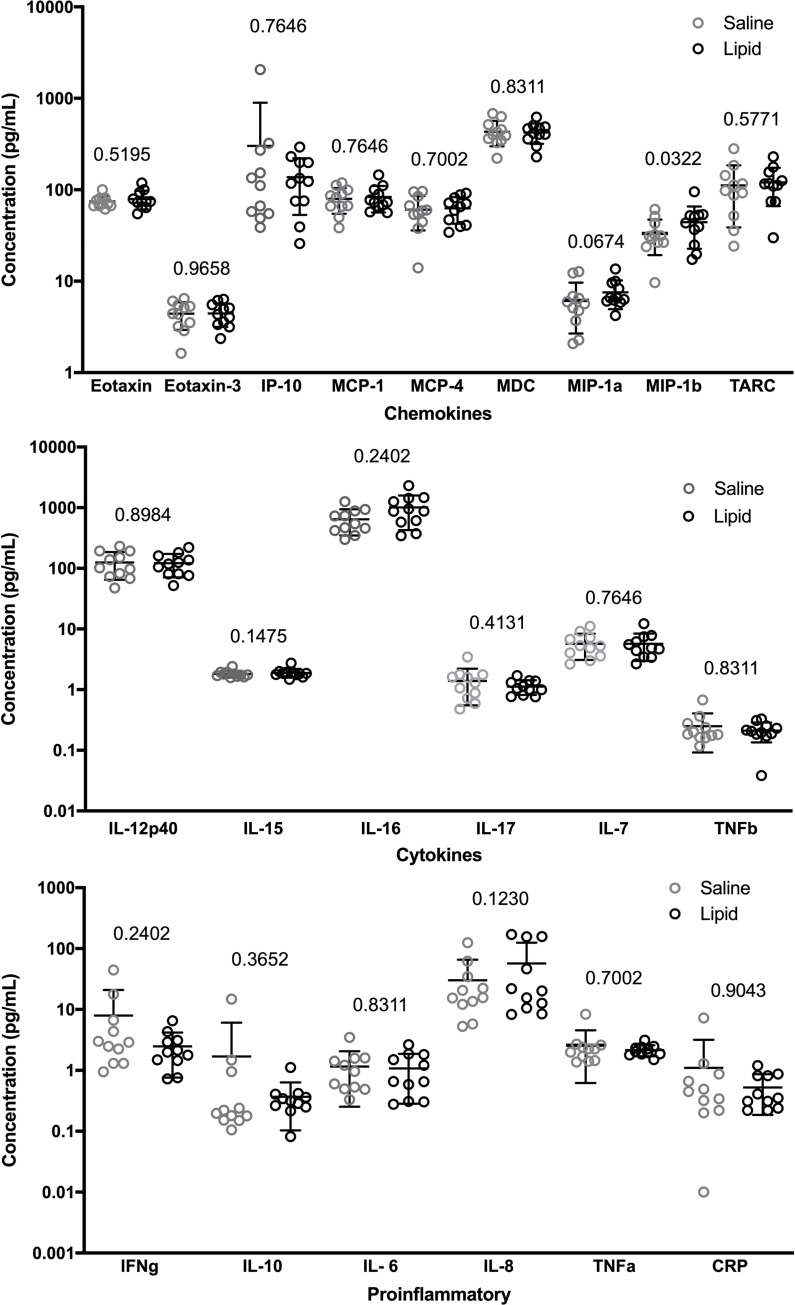
The Effect of Insulin + Fatty Acid Infusion on A) Chemokines, B) Cytokines and C) Proinflammatory molecules. Serum levels were determined by V-Plex human cytokine 30-plex immunoassay or ELISA as described in Methods. Numbers indicate p values, *p<0.05 (N = 11).

Serum profiles are categorized according to the assay specifications, as chemokines (Fig 3A), cytokines (Fig 3B) and proinflammatory molecules (Fig 3C). There were few significant differences observed between the control and lipid/insulin treated arms of the study, in a paired analysis with each participant serving as her own control. All participants remained normotensive during both infusions and blood pressure (BP) values were not significantly different between study visits or infusions. Mean systolic BP was 110 ± 9 for saline and 114 ± 7 for insulin and lipid infusions and diastolic BP 72 ± 9 and 69 ± 9, respectively.

For 9 of the 30 markers of inflammation measured (IL-1, IL-2, IL-4, IL-5, IL-8, IL-12p70, IL-13, GM-CSF and VEGF) a proportion of the participants exhibited analyte levels below the limit of detection, which precluded inclusion in the statistical analysis. However, as shown in Fig 3, we observed no detectable difference, or systematic direction of change, with respect to saline or insulin and lipid infusions.

### Chemokines

A small but statistically significant difference in MIP-1β (CCL4) was detected, with a mean of 1.60 in the experimental arm compared to 1.48 in the control arm (p = 0.032). The related macrophage inflammatory protein, MIP-1α, showed a similar increase in response to infusion of lipid and insulin, which approached statistical significance (p = 0.07).

### Cytokines

There were no statistically significant differences in any of the cytokines measured when comparing the control to the experimental condition.

### Proinflammatory molecules

Again, no statistically significant differences in serum levels were observed comparing control to experimental conditions for any of the proinflammatory molecules measured.

**Fig 4:**
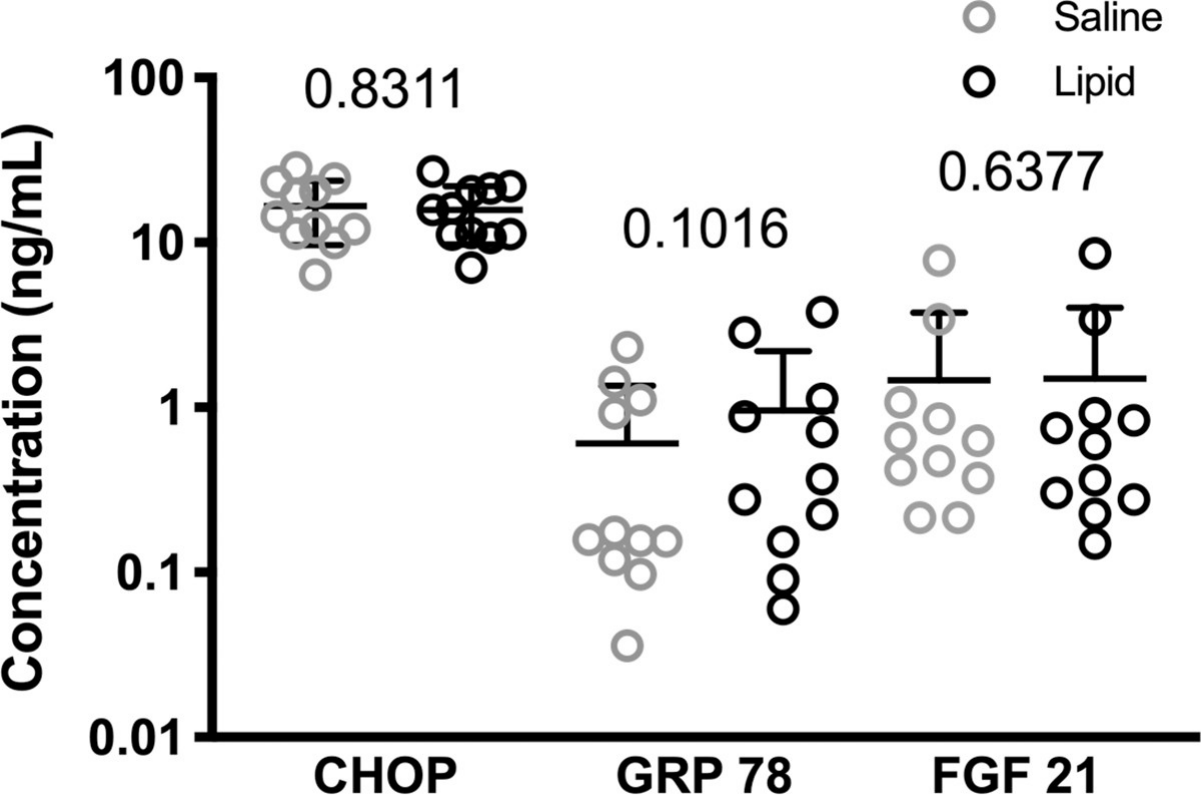
The Effect of Insulin + Fatty Acid Infusion on ER Stress Markers and FGF-21. Serum CHOP, GRP78 and FGF-21levels were determined by immunoassay as described in Methods. Numbers indicate p values (N = 11).

Analysis of the ER stress markers CHOP and GRP78 also showed no significant differences between control and lipid/insulin levels. Similarly, no changes in levels of FGF-21, a proposed regulator of glucose and lipid metabolism were detected (Fig 4).

## Discussion

In this study, we demonstrate that a combination of acutely increased circulating free fatty acids plus insulin, under euglycemic conditions, does not lead to an overall increase in inflammatory signaling in normal weight women, despite reductions in serum LH and FSH, and their response to GnRH, achieved by this intervention (Santoro, *et al* Endocrine Society 2018, Abstract 3399). In the context of obesity, evidence suggests that, while ovarian function may be impaired, the predominant effect is to suppress basal and GnRH-stimulated synthesis and secretion of LH and FSH from the pituitary [3, 4, 14]. We and others have suggested that these effects and the resultant reproductive dysfunction, may be mediated by circulating inflammatory factors that are implicated in obesity [4, 7, 8, 15]. We have previously shown that acute hyperinsulinemia and hyperlipidemia, characteristic of metabolic syndrome, significantly decreases LH and FSH in normal weight women, providing a possible mechanism underlying the hypergonadotropic hypogonadism of obesity [6]. Others have shown that a very similar infusion of non-esterified fatty acids and insulin in lean research participants can result in insulin resistance and elevated TNF-alpha, although circulating cytokines were studied only after 48 hours [16]. Use of a multiplex cytokine array allowed profiling of a comprehensive panel of diverse markers of inflammation, given the limited serum samples available. In our study, although gonadotropin secretion was disrupted within 4 hours, it was not accompanied by changes in circulating inflammatory markers and/or cytokines. Several inflammatory cytokines IL-1, IL-2, IL-4, IL-5, IL-8, IL-12 and IL-13) were below the limit of detection in a fraction (up to 5) of participants, in one or both study arms. These analytes are typically included in clinical panels diagnostic of chronic inflammatory conditions and would have been readily detected if present at or above reference values, indicative of an inflammatory response. We did observe a slight increase in MIP-1β (CCL4) in response to insulin and lipid infusion. MIP-1β is an inflammatory signal and monocyte chemoattractant/ macrophage activator, implicated in inflammatory processes, such as wound healing and folliculogenesis [17]. In young males who are hypogonadal, high levels of MIP-1β are strongly associated with low levels of testosterone and subfertility [18]. MIP-1β is increased in response to palmitate [19] and levels correlate with metabolic inflammation induced by a high fat diet [20]. However, our overall findings imply that there was no acute inflammatory response induced by the experimental conditions and the observed isolated increase in MIP-1β may not be clinically significant. No significant changes were observed in levels of other markers of macrophage activation or polarization [21, 22] (interferon γ, TNFs, interleukins, eotaxin or MIP-1α).

Our previous work has shown that women with obesity had significantly higher baseline IL-6, IL-10, TGF-β, and IL-12 compared with NWW. However, consistent with the observations herein, treatment with omega-3 fatty acids decreased these inflammatory cytokines in women with obesity, but had no effect on serum or urinary levels of FSH, LH or ovarian steroids [12, 15]. In addition, recapitulation of reprometabolic syndrome, by infusion of lipid and insulin, selectively impaired gonadotroph secretion of LH and FSH, indicating a cell specific effect rather than an overall response of the pituitary to systemic inflammation [23]. We have also excluded heparin as a potential factor in decreased gonadotropin levels [24].

ER stress has been implicated in the pathogenesis of obesity [25] and may modulate pituitary gonadotropin secretion and ovarian function [26, 27]. However, we did not observe an acute change in the secreted ER stress markers, CHOP and GRP78 [28]. We previously observed no significant differences in CHOP or GRP78 between women with obesity or of normal weight and these ER stress markers did not correlate with changes in gonadotropin levels [12]. We acknowledge that serum levels of CHOP and GRP78 may not reflect tissue-specific ER stress in the pituitary and therefore cannot be excluded as mediators of gonadotropin suppression. We have also ruled out a global pituitary effect of the insulin and free fatty acid infusion, as other pituitary hormones and their downstream products were unaffected by the infusion [29].

FGF-21 is a regulator of glucose and lipid metabolism with increased levels in obesity [30], which has been implicated in neuroendocrine control of female reproduction. Mice overexpressing FGF-21 exhibit hypogonadotropic hypogonadism [31]. However, other studies show a positive correlation of FGF-21 and LH levels [32] and suggest that modulation of gonadotropins may be an indirect consequence of FGF-21 metabolic effects [33]. Decreases in LH and FSH in this study were not associated with significant changes in FGF-21 levels.

The observed decrease in gonadotrophin secretion could still be explained by a yet unmeasured cytokine-mediated effect on the pituitary or hypothalamus. Although we examined a comprehensive panel of obesity associated cytokines, there are other inflammatory markers that were not measured in this study, including ghrelin, neuropeptide Y, and the recently emerging advanced glycation end products (AGEs) [34]. The acute phase reactant, CRP was not elevated after free fatty acid plus insulin infusion, thus ruling out chronic ambient inflammatory environment in the subjects tested. In the absence of overt systemic inflammation, the observed decrease in gonadotrophin secretion, characteristic of reprometabolic syndrome, may be a direct effect of insulin and/or fatty acids on the pituitary. Very high, supraphysiologic doses of the monounsaturated omega-9 fatty acid oleate induced cellular stress and increased production of reactive oxygen species in a mouse gonadotrope cell line, while also suppressing gonadotropin secretion in response to pulsatile stimulation by GnRH [27]. In a prior study, treatment with omega 3 poly-unsaturated fatty acids reduced both baseline and GnRH-stimulated serum FSH levels in NWW [12, 15].

Strengths of this study include the large number of inflammatory markers we measured, crossover-design of the study, and well-controlled experimental conditions. Weaknesses include the possibility that a critical cytokine was not measure, the sample size is low and, as this was an exploratory secondary analysis and the power of this study was not determined in advance. The findings of this study are likely applicable to healthy, reproductive age women but generalizability may be limited by the largely white study subjects.

In summary, we demonstrate that the combination of acutely increased circulating free fatty acids plus insulin, under euglycemic, hyperinsulinemic conditions, reduces basal and stimulated gonadotrophin secretion but does not lead to a detectable increase in inflammatory signaling in normal weight women. Therefore, the observed decrease in basal and GnRH-induced gonadotrophin secretion associated with the infusion is likely mediated by other factors and is perhaps a direct effect of insulin and/or free fatty acid on the pituitary.

## Supporting information

S1 Checklist (DOC)

S1 File (DOCX)
