# Supplementary material for: A randomised trial examining inflammatory signaling in acutely induced hyperinsulinemia and hyperlipidemia in normal weight women-the reprometabolic syndrome
Source: PLoS One. 2021 Mar 25;16(3):e0247638. doi: 10.1371/journal.pone.0247638 (PMC7993783; doi:10.1371/journal.pone.0247638)
Supplement: S1 File — (DOCX) [file pone.0247638.s002.docx]

**Version Date: 20-December-2019**

1. **Hypotheses and Specific Aims:**

Before any of the well-known adverse effects in pregnancy^2,3^, obesity causes a relatively hypogonadotropic hypogonadal phenotype. Reduced LH, FSH, estradiol (E2) and progesterone secretion are well documented during the menstrual cycles of obese women compared to normal weight women (NWW).^4,5^. Decreased gonadotropin secretion associated with obesity is related to reduced pituitary sensitivity to GnRH^6^. This reduction in pituitary sensitivity suggests mediation by ***circulating factors*** such as cytokines, insulin, or other pro-inflammatory signals known to be elevated in obesity. We have recently discovered that the combination of hyperinsulinemia and circulating free fatty acids (FFAs), but neither agent alone, can acutely decrease gonadotropin secretion in NWW as well as men, establishing a direct causal linkage for the central hypothesis of this proposal: that ***chronic pituitary suppression partially mediates obesity related subfertility***. Our working model is that the combination of excess, possibly pro-inflammatory (omega-6) circulating FFAs and insulin resistance associated with obesity, cause decreased pituitary sensitivity to GnRH, with a resulting relative sex steroid deficit that further exacerbates the obese phenotype. We have named this phenotype the reprometabolic syndrome.

We propose to examine the interrelationships among obesity, reproductive dysfunction and metabolic dysfunction in a mechanistic fashion. We will induce the hypogonadotropic hypogonadal phenotype of obesity in NWW, who will be primed with a high-fat diet (HFD) designed to increase circulating FFAs and produce short-term insulin resistance and higher insulin levels.^1,7-11^ Before and after priming, we will test the additive effects of lipid excess, insulin, and inflammation on the reproductive and metabolic axes.

***Specific Aim 1*** will reproduce the reproductive phenotype of obesity in NWW by: a. infusing insulin and free FFAs in short term experiments and measuring gonadotropin pulsatility and pituitary GnRH response; and b. administering a eucaloric diet that is relatively high in pro-inflammatory omega-6 fatty acids and low in anti-inflammatory omega-3 fatty acids (high fat diet; HFD) for one month while monitoring gonadotropin pulsatility and daily urinary reproductive hormone excretion. A high-fidelity method will be derived to recreate the reprometabolic syndrome.

***Specific Aim 2*** will determine the metabolic changes effected by temporary induction of the reprometabolic syndrome in Aim 1B by examining the anti-lipolytic (low dose) and glucoregulatory (medium dose) actions of insulin with a 2-stage, insulin infusion before and after the HFD is consumed (HFD intervention lasts for approximately one menstrual cycle).

***Specific Aim 3*** will examine the inflammatory milieu that accompanies successful induction of the reprometabolic syndrome in Aim 1B, seeking to identify candidate inflammatory modulators that contribute to the reproductive phenotype.

These studies will be the first to link the reproductive hormonal consequences of chronic high-fat feeding and insulin resistance (hyperinsulinemia) to its potential metabolic outcomes. Taken together, our work will provide new and useful information to design targeted interventions to optimize reproductive potential in obese women, as behavioral interventions have not demonstrated uniform success and may be insufficient^12-14^. This approach may ultimately be mobilized to assist the prevention and avoidance of transgenerational transmission of the adverse ‘reprometabolic’ phenotype.

**Figure 1**. The overall hypothesis and strategy of this proposal is explained in the two graphics above. The basic thesis of this proposal is that the relatively high fat, hyperinsulinemic and secondarily pro-inflammatory milieu of obesity impairs pituitary function. The upper panel explains the strategy to induce the reproductive axis deficit we have observed in obesity (Aim 1), which will demonstrate how an HFD (or a variant thereof) can reliably cause insulin resistance (quantified in Aim 2; lower panel) and whether and how peripheral inflammation may mediate these reproductive effects (Aim 3, lower panel). ***RMS=reprometabolic syndrome***

1. **Background and Significance:**

Female obesity is highly prevalent, affecting 36% of the population, with 64% being either overweight or obese^15^. ***Pre-obesity and obesity are associated with reproductive dysfunction in a weight-dependent fashion,*** at multiple levels of the reproductive axis. Conception is delayed, and fecundability is reduced by 3% per increase in BMI unit above 25 kg/m^2 2^. Among 7,327 pregnancies, obtained from a population-based sample of 55,000 women attempting pregnancy, being overweight was associated with a 16% reduction in the chance of conception per cycle, and obesity was associated with a 34% reduction^2^. The magnitude of the weight-related reduced fecundity was unaffected by the presence of normal menstrual cycle lengths of 27-29 days. Unfortunately, times to pregnancy of 12 months or longer were censored from the data in this study, as it was expected that couples with infertility of this duration would be likely to seek treatment. We have demonstrated similar negative relationships with increasing body mass index relating to longer times to pregnancy^16^ and excess risk of nulligravidity and nulliparity^17^.

In addition to difficulty conceiving^17,18^, obesity has other reproductive effects that lead to poor pregnancy outcomes, and adverse transgenerational outcomes for the offspring^19,20^. Obese women undergoing in vitro fertilization have been reported to have a lower yield of oocytes^21^, reduced fertilization, and lower implantation and live birth rates compared to normal weight women; although others do not note negative effects of obesity on ovarian stimulation, pregnancy and live birth rates when high-intensity assisted reproduction is used (and therefore presumably overrides the reproductive deficit)^22,23^. Higher cycle cancellation rates, spontaneous pregnancy loss^3^ and obstetrical complications^24^, with an increased likelihood of operative delivery^12^, have also been reported in obese women compared to normal weight women. When embryos arising from ova of normal weight women are transferred into obese women, pregnancy rates are improved compared to use of an autologous oocyte, implying that obesity is deleterious at the earliest stages of follicular growth and maturation^25^. Once pregnant, obese women are at substantially greater risk of having children who themselves are prone to obesity, type 2 diabetes mellitus, and dysmetabolism^26^. Whether this transgenerational transmission of obesity occurs from metabolic programming during pregnancy, or whether it occurs at or prior to conception, due to the impacts on the maternal hypothalamic pituitary gonadal axis, is currently unknown. Reproductive deficits at the level of the hypothalamus, pituitary, gonad and uterus have all been proposed as mediators of the adverse reproductive outcomes of obesity. This proposal is specifically focused on the contribution of the pituitary-ovarian axis to the problem.

**Through a series of NIH funded studies, we have generated exciting preliminary data that 1) confirms the relatively hypogonadotropic hypogonadal phenotype of simple (i.e., not associated with polycystic ovary syndrome, which affects 5-7% of the adult population) obesity; 2) localizes the defect to the pituitary gland, and 3) identifies free fatty acids and insulin, acting in concert, as the potential cause of what we have named the ‘reprometabolic syndrome’.** In the first series of studies, which have been published, we confirmed inadequate luteal progesterone excretion and markedly reduced LH pulse amplitude in the follicular phase of menstrual cycles of morbidly obese but cycling women, prior to undergoing bariatric surgery (Figure 2)^1^. In follow-up studies, also published, we observed an improvement in the reproductive phenotype after surgery and weight restabilization at a level reflecting approximately 25% loss of starting body weight (Table 1)^7^. In these post-weight loss cycles, progesterone excretion and LH pulse amplitude had partially recovered. The pre-surgery BMI of the study participants was 47.3 +/- 5.2 kg/m^2^. After weight loss, the mean BMI dropped to 32.0 +/- 2.9 kg/m^2^; despite the dramatic weight loss, the women remained obese. These data imply but do not prove that weight loss could restore a normal reproductive phenotype.

| **Parameter/Group** | **Pre-Op (N=23)** | **Post-Op (N=6)** | ***P*-value** |
| --- | --- | --- | --- |
| BMI (kg/m^2^) | 47.3 +/- 5.2 | 32+/-2.9 | <0.01 |
| Cycle Length (days) | 29.5 +/- 4 | 27.2 +/-2.8 | 0.2 |
| Peak LH (mIU/mg Cr) | 27.6 +/- 12.4 | 43.7+/-10.6 | 0.007 |
| LH AUC (mIU/mg Cr) | 168.8 +/- 24.2 | 292.1+/-79.6 | <0.0001 |
| FSH AUC (mIU/mg Cr) | 22+/-23 | 36.2+/-16.7 | 0.17 |
| Luteal Pdg AUC (ug/mgCr) | 32.8 +/- 10.9 | 73.7 +/- 30.5 | <0.001 |

**Table 1**: From Rochester, et al^7^. Pre and post-operative menstrual cycle parameters in morbidly obese women undergoing bariatric surgery (primarily gastric reduction). LH and Pdg excretion rose appreciably after surgery both in terms of peak level on the day of the LH surge (LH peak) and integrated, whole cycle LH (LH AUC). Data shown are mean +/- SEM. Findings were identical, with the same categories of statistical significance, when paired observations only were used (i.e., when the 6 women who had an ovulatory postoperative cycle were compared, using paired analysis, to their pre-operative cycles).

A luteal phase defect type of progesterone (serum) or Pdg (urine) pattern has been consistently observed in cross sectional studies of women with obesity, including some with very large sample sizes in the hundreds^4,5,28^, suggesting that this is a consistent manifestation of the obese reproductive phenotype, and is related to the subfertility. Available data suggest that rapid weight loss experimental models have limitations in their ability to explain the pathophysiology7^,27,29^. Therefore, we have chosen to directly test causation by attempting ***to create the reproductive deficits associated with obesity in normal weight women (NWW).***

| **Group/Parameter** | **LH amplitude** | **LH frequency** | **BMI** |
| --- | --- | --- | --- |
| NWW | 1.59 +/- 1.5 | 7.3 +/-0.5 | 21.3 |
| Obese Pre-Op | 0.85+/- 0.1 | 7.8 +/-0.9 | 47.9 +/- 5.2 |
| Obese Post-Op | 1.12+/-0.2 | 7+/-0.4 | 32+/-2.9 |
| P value, pre vs post | 0.01 | NS | <0.01 |

**Table 2:** Pre and Post-bariatric surgery LH pulsatility parameters compared to normal weight controls. BMI is shown as mean +/-SD, pulse parameters shown as mean +/-SEM. The number of observations used for statistical analysis is 12 for normal weight, 8 for high BMI pre-op. 4 women completed both pre and post op assessments; cross sectional analyses also yielded statistically significant results. Results were verified using two objective pulse detection algorithms. LH pulse frequency did not differ between any of the groups, whereas LH pulse amplitude was reduced by about half in the obese women pre-operatively, with a return towards normal after surgery.^5^

To further examine the reprometabolic phenotype, we performed 12-hour, frequent blood sampling studies to assess LH pulsatility. Profound suppression of LH pulse amplitude was observed in cross sectional study of obese compared to NWW, and after successful bariatric surgery (defined above), LH pulsatility returned towards normal but did not completely normalize (Table 2).

**Possible underlying causes for the relative hypogonadotropic hypogonadism of obesity.** Insufficient central GnRH stimulation, insufficient pituitary production of LH and FSH, and/or inadequate end-organ (follicle or corpus luteum) response are all potential pathways that can lead to the inadequate luteal phase phenotype that we and others have identified and characterized. ***We have systematically tested these possibilities and identified reduced pituitary responsiveness to GnRH (Figure 3) in obese women as a consistent neuroendocrine defect.***


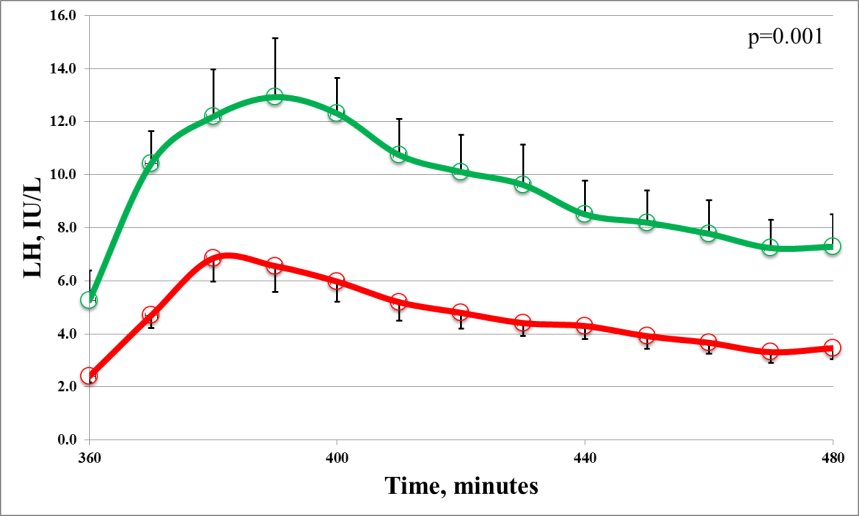


**Figure 3**. Response to a 75 ng/kg bolus

of GnRH in a group of 10 obese Women

compared to 10 NWW sampled in the

follicular phase of the menstrual cycle.

P value for area under the curve is

0.001

We examined the LH response to exogenous GnRH, along with gonadotropin clearance and the progesterone response to exogenous LH. In the follicular phase of the menstrual cycle, obese women were found to have about half the NWW’s LH response to a submaximal, physiologic-range dose of GnRH (75 ng/kg; Figure 3). In the luteal phase of the menstrual cycle, obese and NWW did not have a significantly different response,^6^ largely because the NWWs had more variable responses to exogenous GnRH, which obscured between-group differences. ***These findings indicate that pituitary responsiveness to exogenous GnRH is reduced in obese women at the time in the menstrual cycle most critical for optimal folliculogenesis and corpus luteum health. This reduction in pituitary responsiveness is likely at least partially responsible for the reproductive alterations we have observed***. We have also rigorously tested the pharmacokinetics of exogenous recombinant LH and endogenous LH disappearance, and found no differences in clearance of LH between NWW and obese women that might account for their lower circulating gonadotropins^5^. Systematic examination of the progesterone response to LH in the luteal phase has preliminarily shown no differences between obese and normal weight women; there does not appear to be selective impairment of the corpus luteum response to endogenous LH. Our studies cannot rule out an ovarian or oocyte deficit directly imposed by obesity; this is neither the focus of the current application nor is it directly accessible to us in human studies at this time.

**Potential mediation of HPO axis suppression in obesity—the ‘reprometabolic syndrome’**. Abiding features of obesity are hyperlipidemia, insulin resistance and local (adipose) as well as systemic inflammation. We have found evidence for a combined effect of hyperinsulinemia and high circulating free fatty acids (FFAs) in acutely suppressing gonadotropin secretion in both men and women subjected to fatty acid infusion during a hyperinsulinemic, euglycemic clamp (women shown in Figure 4). Either FFAs or insulin alone did not suppress gonadotropins. This short-term experiment suggests that we are on the right track in our selection of candidate circulating factors as effectors of suppression of the HPO axis in obesity. Exposure to high dietary fat—particularly saturated fat—directly impairs cell membrane function and could cause pituitary dysfunction apart from altering the lipid profile^34^. High dietary fat is also widely implicated as a cause of insulin resistance and dysglycemia^34^.

**Figure 4**. Rapid induction of reprometabolic syndrome in normal weight women is achieved over 6 hours by infusion of both free fatty acids (Liposyn II or Intralipid) and insulin together. Note that both LH and FSH are suppressed. Data are shown for 10 regularly cycling, normal weight women (mean BMI 22.8 [19.3-28]) of reproductive age (mean 32.5 [22-42]). A crossover design was employed such that all women received each of the following infusions; control (saline), 20% lipid emulsion/heparin (45ml/h), insulin (40 µg/kg/min) under euglycemic clamp conditions, and a combination of lipid + insulin, over a series of 4 visits. Blood was collected every 2 hours and LH and FSH measured by a direct chemiluminescent assay (Perkin Elmer; Autodelfia). Transverse means were compared between control and treatments for each woman using a paired t test. Means are shown with standard error bars. SHBG and E2 did not differ between any of the treatment groups. Induction of insulin resistance and elevation of plasma non esterified fatty acids (NEFAs) were confirmed in all participants who received both insulin and lipid infusion. Similar data (not shown) has been obtained in men

Obesity is highly prevalent and has major implications for reproduction. An improved understanding of how metabolism and reproduction interact in obesity is critical for the development of targeted and effective treatments. We shall perform critical, physiologic probing of the problem that can be adapted over the long term towards the development of treatments either to facilitate weight loss or to promote healthy reproductive hormone secretion in obese women to abrogate the negative, epigenetic downstream consequences to their offspring. This is particularly important in the preconceptual obese woman, as accruing evidence suggests that lifestyle intervention programs in obese women who are already pregnant do not decrease the impact of transgenerational adverse metabolic programming^14^

1. **Research Methods**

Our overall analytical approach to all aims is shown below in Figure 5.

| Aims 1 and 2. Establish the causal effect of components of obesity on RMS  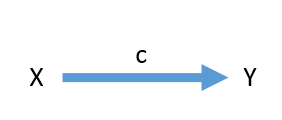  Where:  X (obesity) is the causal variable,  Y (RMS) is the outcome.  X is assumed to *cause* Y.  Path c is the total effect of obesity on RMS. | Aim 3. Evaluate inflammation-related cytokines as potential mediators of the relationship between FFAs and RMS.  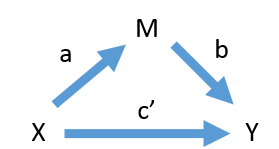  The effect of obesity on RMS may be mediated by inflammation (shown as M above) Path c’ is called the direct effect. |
| --- | --- |
| X = the obesity phenotype, quantified by hyperinsulinemia and elevated FFAs  Y = Reproductive Metabolic Health, quantified by LH pulse amplitude | The direct effect of obesity on RMS will be assessed within the framework of the mediation hypothesis. |

**Figure 5.** Analytic strategy as it relates to the aims. In Aims 1 and 2 we will determine whether select components of obesity can cause the reprometabolic syndrome (c shown to indicate causation). In Aim 3, we will determine whether inflammatory cytokines mediate this process.

***Outcome Measures:***

***Aim 1:***

The experimental strategy will utilize the classical endocrine approach of inducing the hypothesized defect (reprometabolic syndrome) with the goal of developing a feasible, high-fidelity model. A combination of hyperinsulinemia and elevated FFAs will be examined to determine if the previously reported elements of the reprometabolic syndrome (reduced LH pulse amplitude and lower FSH levels) can be faithfully created.

***Aim 1 Primary outcome*:** The primary outcome for Aim 1 will be LH pulse amplitude as assessed in the non-intervention frequent sampling study and compared to the early follicular phase study under treatment (i.e., either insulin + FFAs for Aim 1A or HFD for Aim 1B). LH pulsatility will be analyzed using a modification of the original Santen and Bardin method, as previously described and utilized by the PI in numerous studies^1,6^

***Aim 1 Secondary outcomes:***

1. GnRH response will be compared between the non-intervention and intervention study as described above for gonadotropin pulsatility. We have used area under the curve methods to determine the LH response to exogenous GnRH and will utilize the same methodology as we have done in the past.^6^
2. FSH parameters will be compared between the non-intervention and intervention studies for both aims as described above for gonadotropin pulsatility. We will compare mean FSH, as pulsatility of FSH is less obvious than LH.
3. We will compare changes in gonadotropin pulse frequency (for LH, and if we can detect distinct FSH pulses, we will compare FSH as well), mean LH and FSH and kinetics of LH, and if possible, FSH, before and after the intervention, as previously reported^7,32^.
4. For Aim 1B, urinary hormone profiles will be assessed for the entire cycle before and two cycles after initiation of the HFD using previously described menstrual cycle parameters suitable for urinary hormone determinations^42^. The presumptive day of ovulation, called the Day of Luteal Transition (DLT) will be determined for all cycles that demonstrate a Pdg increment consistent with ovulation^42^. Follicular and luteal phase lengths will be calculated, as will integrated follicular, luteal and whole cycle LH, FSH, E1c and Pdg^4,42^. Whole-cycle parameters will be calculated and compared before and after HFD using a paired t-test for interval data which can be reasonably fit by a linear model framework, and in a linear regression model if adjustment for covariates is necessary. No statistical adjustment will be made for multiple comparisons of potentially covarying hormones. Hormones measured in daily urine will be modeled using linear mixed-effects regression to account for repeated measures nature of the data. Right-skewed variables will be analyzed on the log-scale, back-transformed and reported as geometric mean and 95% CI.
5. We will also measure ovarian reserve markers: anti-Mullerian hormone (AMH; Ansh Laboratories), inhibin A and B (DSL, Webster, TX) before and after the intervention to determine whether they are altered by the intervention. All of these hormones have been previously measured by the PI^43,44^.
6. Activity will be monitored monthly during the entire study, and weekly during the month of HFD in all participants. The baseline cycle of collection will be used as the participant’s benchmark and a deviation of >10% from baseline activity in any given week will alert the study staff to counsel the participant to maintain her usual level of activity.

***Aim 2:***

We will determine the metabolic consequences of the induction of the reprometabolic phenotype with HFD(Aim 1b) by assessing the glucoregulatory and anti-lipolytic actions of insulin with a 2-stage, hyperinsulinemic, euglycemic clamp (HEC) to evaluate both suppression of lipolysis (low dose: 4 mU/m2/min) and suppression of hepatic glucose production (40 mU/m2/min).

***Aim 2 Primary outcome***: Our primary outcome will be M, which represents the steady state amount of glucose metabolized at the set insulin infusion rate under euglycemic conditions, which is equal to the glucose infused when the participant is euglycemic during the second stage of the HEC^49^. The final 30 minutes of the clamp period will be considered steady state. Glucose concentrations will be determined with the glucose oxidase method (Beckman Glucose Analyzer 2; Beckman Instruments, Fullerton, CA), while ELISA methods will be used for insulin measurements (Alpco, Salem, NH).

***Aim 2 Secondary outcomes:***

1. *Insulin, glucose and* FFAs will be measured by the CTRC laboratories. Plasma non-esterified FFAs will be measured after lipid extraction of plasma (Wako Diagnostics, Richmond, VA). Lipids are measured by enzymatic methods (Quest Diagnostics- Nichols Institute, Chantilly, VA). Ketones will be measure by the study personnel using a urinary strip on discarded urine used for pregnancy test.
2. RBC lipids will also be compared, as we predict that the HFD will result in increased omega-6 rich FFAs and less omega-3 FFAs.
3. *DEXA body composition* will be measured before and after the intervention. Dr. Wendy Kohrt will collaborate with the PI, as she has in the past, on these measurements^6^.
4. Weight will be monitored weekly to assure that participants are not losing or gaining weight, which could affect study results. A weight gain or loss of more than 1.5lbs in one week will alert the study staff to counsel the participant and/or adjust the HFD food modules.

***Aim 3:***

We will examine the inflammatory milieu that accompanies successful induction of the reprometabolic syndrome in Aim 1, seeking to identify candidate inflammatory modulators that may specifically contribute to the phenotype.

***Description of Population to be Enrolled:***

We will recruit up to 60 normal weight, normally cycling women aged 18-38, who will meet the criteria below:

Inclusion Criteria:

- BMI at least 18 but less than 25 kg/m^2^
- No history of chronic disease affecting hormone production, metabolism or clearance
- No use of medications known to alter or interact with reproductive hormones or insulin metabolism (e.g. thiazolindinediones, metformin)
- No use of reproductive hormones within 3 months of enrollment
- Normal prolactin and thyroid stimulating hormone levels at screening
- History of regular menstrual cycles every 25-35 days
- Use of a reliable method of contraception (female or male partner sterilization; IUD; abstinence; diaphragm)
- Normal hemoglobin A1c
- Screening hemoglobin >11gm/dl

Exclusion criteria:

- Women with a baseline dietary assessment indicative of >40% daily calorie consumption from fat (as calculated based upon initial 3 day diet record) will be excluded, as the impact of increasing their dietary fat intake may be minimal.
- Women with current or a history of diabetes, prediabetes, or gestational diabetes.
- Women with fasting triglycerides >300mg/dl at screening will be excluded, as they might be at risk for acute elevation of triglycerides and even pancreatitis if placed on a high fat diet.
- Inability to comply with the protocol. Individuals who travel frequently, or who eat most of their meals outside of their home will be excluded, as it will be difficult to impossible for them to comply with the diet, to pick up the food cartons, etc.
- Because high proportions of dairy fat will be needed to attain 48% calories from fat in the diet, vegans and lactose intolerant individuals will be excluded.
- Pregnant women or women planning to become pregnant will be excluded.

***Study Design and Research Methods***

Consent/Screening Process:

A script for screening participants by telephone and by webisite survey who inquire in response to advertisements will be used, which will include the required verbal consent language. Participants who are eligible for enrollment will be asked to come to the Clinical and Translational Research Center (CTRC) for an intake examination and qualifying laboratory tests. At this time, informed consent is obtained by the PI, the Professional Research Assistant, or an IRB approved designee, according to the scripted process. The informed consent discussion is always held in a private room that is free of distractions and participants are allowed the opportunity to ask questions and have their questions answered to their satisfaction before signing the informed consent document. Once informed consent has been provided, baseline visit procedures are performed as outlined in the procedures section.

Study procedures:

For Aim 1a we will enroll 12 normal weight women to complete the study. For Aim 1b, we will enroll 20 normal weight women which may include women who participated in Aim 1A but not necessarily so.

Aim 1A will reproduce the reproductive phenotype of obesity in normal weight women by: infusing insulin and free FFAs in short term experiments and measuring gonadotropin pulsatility and pituitary GnRH response

Aim 1B will induce a chronic model of the reprometabolic syndrome by administering a eucaloric high fat diet for one month while monitoring gonadotropin pulsatility and daily urinary reproductive hormone excretion. In Aim 1b we want to demonstrate that a eucaloric high fat diet can induce insulin resistance and/or elements of the reprometabolic syndrome in women.

Aims 2 and 3 will be an exention of analysis from the procedures performed and samples collected in Aims 1.A and 1.B. They will not require additional enrollment of study subjects or additional procedures.

**Pilot**: consisted of the Principle Investigator testing out the proposed diet invervention for Aim 1.B. The test diet was given for two weeks. There were no subjects consented or enrolled, and the Investigator declined compensation.

**Aim 1A:** will consist of four visits: a screening visit (12 hour fast required) and three study visits. During the screening visit participants will be provided with instructions and supplies to collect their daily morning urine and to use home ovulation predictor kits. Determining the day of ovulation will allow us to better predict the timing of the following menses (which almost always occurs 12-15 days after the urinary LH surge identified by the ovulation predictor kit). Accurate prediction of cycle Day 1 will permit more efficient scheduling of the frequent sampling studies, which must occur within a window of 1-4 days after the onset of menses (cycle days 2-5). We will ask participants to track their menstrual cycle by recording the day they start their period and the day that they ovulate and notify the study coordinator when evidence of a urinary LH surge has occurred. This will involve dipping a test stick in urine for several days to see if it changes color. Additional ovulation prediction kits will be provided if needed to the participants at the Visit 1 & 2. Participants will be administered saline only (limited subsample), saline + heparin infusion, and an insulin/intralipid/heparin infusion over 6 hours. Each participant will receive at least a saline+heparing infusion and an insulin/intralipid/heparin infusion, but the order in which the treatments are given will be randomly assigned. The insulin/intralipid/heparin infusion will consist of 20% lipid emulsion (45ml/h), heparin (24 units/kg/hr), and insulin (20 mU/m2/min) under euglycemic clamp conditions (maintained by using dextrose). Saline will be infused at a rate of 200 ml/h with heparin (24 units/kg/hr). During the saline only visit, heparin will not be infused. We have added a ‘saline only’ visit to validate the findings of the saline+heparin infusion study and to rule out any independent effect of heparin. At the same time, participants will undergo a 6-hour frequent blood sampling session at each visit. Muscle oxygenation will also be measured before and at 4 hours of both infusions. Partial thromboplastin time (PTT) levels will be followed with heparin infusion adjustments as needed to avoid over-anticoagulation. Each visit will be performed in the early follicular phase of the menstrual cycle and will be completed in consecutive cycles whenever possible, but no longer than within 4 cycles from the first study. Please see table description below:

|  | **Pre-cycle 1** | **Visit 1** | **Visit 2** | **Visit 3** |
| --- | --- | --- | --- | --- |
| Visit# | L0=screen | L1 | L2 | L3 |
| Description/Duration | 2 hours | 6 hours  Concurrent FSS infusion* | 6 hours  Concurrent FSS infusion* | 6 hours  Concurrent FSS infusion* |
| Cycle Day |  | 2-5 | 2-5 | 2-5 |
| History | X |  |  |  |
| Physical | X |  |  |  |
| Pregnancy Test |  | X | X | X |
| Routine safety labs (CBC/CMP) | X | X | X | X |
| Hemoglobin A1C | X |  |  |  |
| Prolactin | X |  |  |  |
| Fasting Lipid Panel | X | X | X | X |
| TSH | X |  |  |  |
| PTT | X | X | X | X |
| FFA | X | X | X | X |
| Informed Consent | X |  |  |  |
| Weight | X | X | X | X |
| Ovulation Prediction Kits | X | X | X |  |
| Frequent blood sampling |  | X | X | X |
| Administration of GnRH |  | X | X | X |
| Muscle Oxygen Measurement |  | X | X | X |
| Infusion* |  | X* | X* | X* |

*participants will receive either an insulin +Iipid + heparin infusion, a heparin + saline (control), or saline only in random order

**Aim 1B:** will consist of 11 in-person visits.

| Screening |
| --- |

**Verbal screening**: At the time of verbal screening for this aim, participants will be provided with a 3 day food diary by email. They will be asked to complete the diary and bring it with them to the screening visit. Participants will also be instructed to fast for 12 hours prior to their in-person screening visit.

**In-person screening**: Participants will be consented and will have a history and physical completed. We will perform a urine pregnancy test, obtain their current medications, their height and weight, a blood sample for screening labs (CBC/CMP, lipid panel), and review their 3-day food diary and food preferences. They will also undergo a DEXA procedure. After examining the results of these tests, we will confirm if the participant meets the inclusion/exclusion criteria. Participants will be provided with instructions and supplies to collect their daily morning urine and to use home ovulation predictor kits. Determining the day of ovulation will allow us to better predict the timing of the following menses (which almost always occurs 12-15 days after the urinary LH surge identified by the ovulation predictor kit). Accurate prediction of cycle Day 1 will permit more efficient scheduling of the frequent sampling studies, which must occur within a window of 1-4 days after the onset of menses (cycle days 2-5). We will ask participants to track their menstrual cycle by recording the day they start their period and the day that they ovulate and notify the study coordinator when evidence of a urinary LH surge has occurred. This will involve dipping a test stick in urine for several days to see if it changes color. At the conclusion of the screening visit or prior to Visit 1.1 we will provide each participant (from August 2019 and on) with a fecal collection kit to bring back to Visit 1.1 to obtain a pre-diet collection.

| Cycle 1 |
| --- |

**Visit 1.1**: Prior to initiating further study procedures, participants will be provided with a Fitbit. They will be asked to use the Fitbit and collect daily morning urine starting on day 1 of their next cycle (Cycle 1) until Visit 8 (the start of Cycle 5). At the beginning of their first cycle (between days 2-4), participants will have a pregnancy test, have their height and weight recorded, and provide a blood sample for safety labs (CBC/CMP) as well as hormones (AMH), cytokines. Ketones will be measured by study personnel using a urinary test strip on discared urine used for the pregnancy test. We are proposing to look at urinary ketones because we want to ensure we are not inducing a ketogenic state in the participants. Participants will undergo a 6-hour frequent blood sampling study (FSS) to examine LH pulsatility and GnRH responsiveness. Gonadotropin pulsatility is assessed by withdrawing 3cc of blood every 10 minutes from an indwelling IV catheter. At 4 hours a physiologic bolus of GnRH 75 ng/kg will be administered to assess pituitary LH response and q10minute sampling will continue for the remaining 2 hours. This is described in the figure below. To avoid wasting any blood, a closed tubing system is used when a nurse is available. Blood is withdrawn into the tubing and the sample is taken without discard from a proximal IV port. When a nurse is not available, a lab technician will use gravity tubing to draw the blood. At the end of the visit a small amount of blood will be drawn for another CBC/CMP. Participants will be provided breakfast and lunch during this visit.

**Data Summary for our 6 in-patient visits (4 clamps, 2 FSS-only)**

| Visit 1.1 Frequent Sampling: LH and FSH every 10 minutes.  Experimental paradigm for Aim 1A and B, indicating the timing of frequent blood sampling study and GnRH dosing for gonadotropins.  GnRH 75/ng kg  *0_____l_____l_____l_____l_____l_____6 hours*  *q 10 minute frequent blood sampling for 6 hours*  *1^st^ 4 hours: endogenous LH pulsatility only (blue); at 4 hours:*  *75ng/kg GnRH given (arrow); sampling completed at 6 hours*  *(shown in red)* |
| --- |
| Visit 1.2 Hyperinsulinemic Euglycemic Clamp  -30 0 30 60 90 120 150 180  -30-0 min: Insulin: Insulin: 40 mU/m^2/^min End of study  Baseline 4 mU/m^2^/min (low dose) (high dose) |

**Visit 1.2**: After the 1.1 visit, the participants will return after fasting overnight. They will have their height and weight recorded. They will provide a blood sample for baseline safety labs (CBC/CMP, lipid panel, FFAs ). Additional blood samples are drawn during the basal period, and Stages 1 and 2 of the HEC to assess insulin, FFAs and glycerol. Participants will have 2 IV lines placed in their arm for infusion of insulin (constant) and 20% dextrose (variable, as needed to maintain euglycemia, defined as a blood glucose of 90-110 mg/dl) and glucose monitoring. A HEC will be performed for 3 hours (180 minutes). Plasma glucose is measured every 5 minutes, with concentrations determined immediately upon collection, and the glucose infusion rate adjusted to maintain euglycemia throughout the HEC. At the end of the visit they will provide another blood sample for safety labs and will be provided with lunch. Participants will be provided a three month supply of iron supplements and be instructed to take one 325mg tablet daily for the next three months.

| Cycle 2 |
| --- |

With the onset of the next menstrual period (Menses #2, start of Cycle #2), a eucaloric, high fat diet (48% energy from fat) will be administered. Participants will continue collection of daily morning first urine, use their Fitbit, and intake of iron supplements over the course of Cycle 2. Also, participants will be asked to use home ovulation predictor kits.

**Visits 2-5**: Participants will be asked to pick up meals twice per week, return their empty food containers, and will be asked if they have any questions about the diet. At the first meal pick-up each week, they will also have their weight recorded, the study team will review their Fitbit information, and they will provide a fasted blood sample for fatty acid analysis. At each of these visits, they will return their collected urine and receive more urine collection supplies. In addition to the aforementioned supplies we will also provide each participant with a second fecal collection kit to obtain a post-diet fecal sample.

| **Cycle 3** |
| --- |

Participants will continue collection of daily morning first urine, use their Fitbit, and intake of iron supplements over the course of Cycle 3.

**Visit 6.1**: At the beginning of their third cycle (between days 1-4), participants will report to the inpatient CTRC. This visit will be identical to visit 1.1 except participants will eat their high fat diet.

**Visit 6.2**: After visit 6.1, and after fasting overnight, the participants will return for a repeat of Visit 1.2.

After Visit 6.2, participants will resume their normal diet.

| **Cycle 4** |
| --- |

Participants will continue collection of daily morning first urine, use their Fitbit, and intake of iron supplements over the course of Cycle 4.

**Visit 7**: At the start of their fourth cycle, participants will have urine pregnancy test and their weight recorded, will return their collected urine, empty iron supplement bottle(s), undergo a DEXA procedure, and the study team will review their Fitbit information with them.

| **Cycle 5** |
| --- |

**Visit 8**: At the beginning (between days 2-5) of the participant’s fifth cycle they will be asked to fast overnight and come in for a visit. They will have a history and physical completed, and we will obtain their current medications, their height and weight, and a blood sample for labs (CBC/CMP, lipid panel). Participants will return their collected urine samples and the study team will review their Fitbit activity. A follow-up email will be sent out with a REDCap link to an anonymous exit survey to better predict pitfalls of the study. In the event that a participant does not see the study to completion, the anonymous survey will be sent to them upon confirmation of withdrawl/elimination.

***
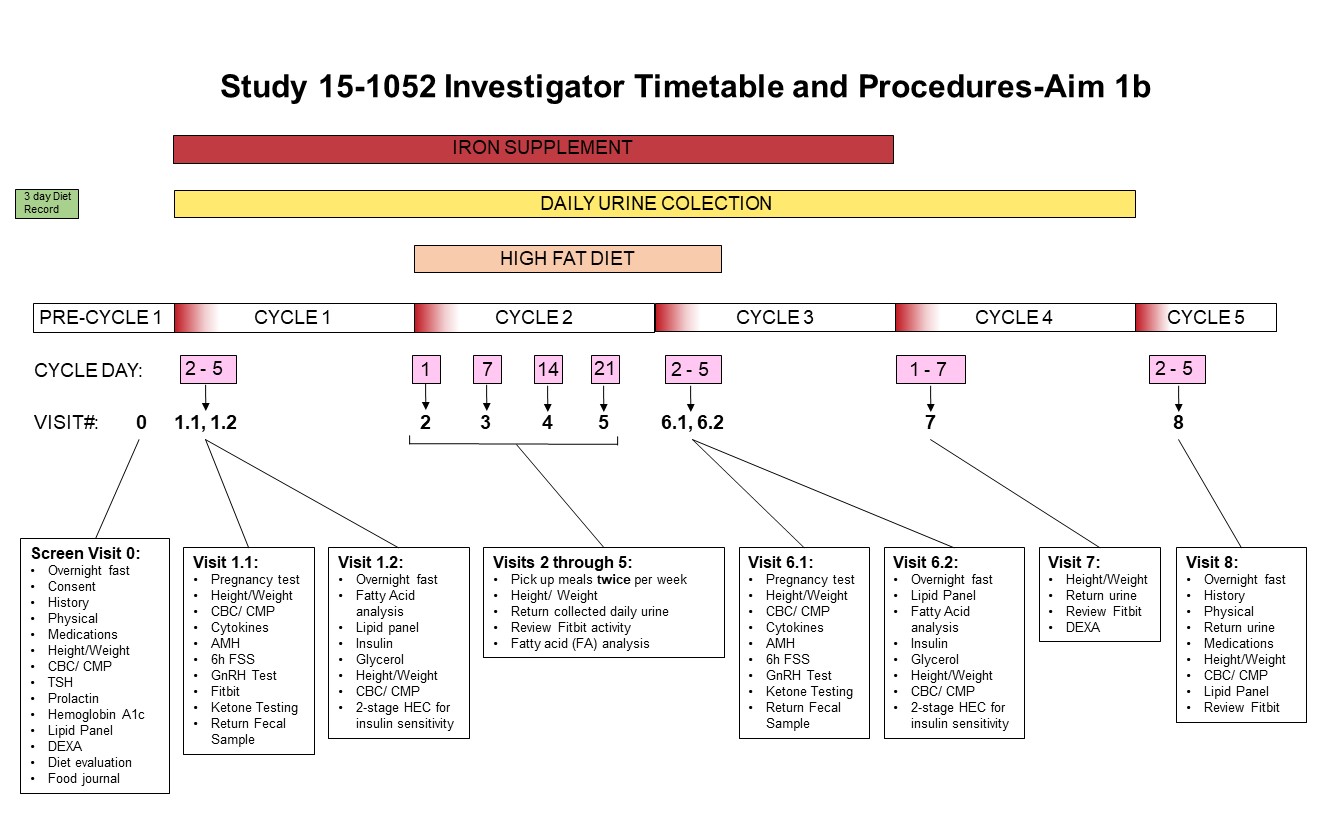
***

***Description, Risks and Justification of Procedures with Data Collection Tools***

**Risks and plan to minimize risks:**

***Risks of a high fat diet****:* it is expected that a 48% energy from fat diet administered for an entire menstrual cycle will not pose a significant risk to participants, as this is within the range of dietary fat consumption among the US population (90th centile; 36). However, we will monitor all participants through 2 recovery cycles to determine whether or not the hormonal deficits associated with obesity are induced and then recover when the diet is withdrawn. While long term consumption of diets that are high in fat have been associated with development of coronary artery disease, these data are by no means well established for women and often relate to the particular type of fat consumed (polyunsaturated versus saturated fat). Reduction in fat consumption for a mean of 8.1 years in the Women’s Health Initiative was not associated with reduced cardiovascular disease ^6-8^. Moreover, a recent study of gestational diabetics administered a diet high in complex carbohydrates and fat (45% calories from fat) for an entire trimester of pregnancy, did not result in any adverse, short-term metabolic outcomes^9^. It therefore seems exceedingly unlikely that an intervention of this degree and duration will have any kind of negative impact. We will monitor reproductive hormones and lipid panels throughout and will repeat these and routine safety labs at the end of the recovery cycle.

***Risks of medications to be administered****:*

***Insulin****:* recombinant human insulin (Humulin) will be administered. Its chief side effect is hypoglycemia. As all participants will be undergoing glucose monitoring every 5 minutes during the clamp studies, their glucose level will be continuously adjusted by adjusting the intravenous dextrose to maintain blood glucose within a 90-100 mg/dl range.

***Intralipid*** *(Aim 1A only):* Intralipid will be administered as an intravenous infusion. Side effects reported for this compound include headache, dizziness, nausea, vomiting, sweating, difficult or labored breathing, or, rarely, anaphylactic type reactions have been reported. Participants will be monitored throughout the infusion in the CTRC, where epinephrine is readily available for any allergic reaction.

***Dextrose (20%):*** will be administered to maintain the blood glucose level between 90-110 mg/dl.

***Heparin****:* will be used in concert with the intralipid infusion to maintain catheter patency and stimulate lipolysis. Adverse reactions to high doses of heparin include hemorrhage and thrombocytopenia. Very long term use (months) has been associated with osteopenia. Heparin will be used at very low doses in this experiment and may induce short term anticoagulation but would not be expected to cause thrombocytompenia or osteopenia at this low dose. Anaphylactic reactions have been reported to occur with heparin, and participants will all be given heparin at the CTRC where nursing personnel monitor them continuously and epinephrine is readily available. In addition:

1) aPTT will be confirmed before participants leave the CTRC to ensure levels are not in excess of the targeted degree of therapeutic anticoagulation in the outpatient setting (aPTT < 120 seconds -- the upper target of therapeutic anticoagulation used in inpatient or outpatient setting).

2) aPTT will be measured during peak infusion (mid-interval) at ~3 hours. The heparin infusion rate will be reduced by 50% if aPTT is > 100, and by 25% (to standard anticoagulation dosing) if the aPTT is 80-100. Levels will be re-measured at 6 hours (toward the end of the infusion) to ensure the aPTT is < 120 secs before allowing the participant to be discharged. If the 6 hour aPTT is > 120, another will be checked at 7 hours, one hour after discontinuation of the heparin (t=7 hours) to ensure that the aPTT is < 120 prior to discharge or on a downward trajectory that ensures aPTT<120 by time of discharge.

3) Patients will be counseled about possible side effects of longer clotting times, behaviors to avoid in the coming hours, what to look for and what steps to take should symptoms develop. Aspirin and Ibuprofen should be avoided for at least 2 hours if the patient is discharged with an abnormal aPTT (>35 seconds).

***GnRH (gonadotropin releasing hormone):*** GnRH is a 10 amino acid peptide that has a short serum half-life and minimal known antigenicity. If given in large doses, it has been associated with a risk of multiple pregnancy. Our participants will all be using contraception and tested for pregnancy at the onset of each assessment.

*Iron Supplements:* Common side effects include an upset stomach, pain, constipation or diarrhea, nausea, or vomiting.

***Risks to pregnant women****:* Although none of the medications listed above are contraindicated in pregnant women, there is no reason for a pregnant woman to be exposed to any of the potential risks above. Our goal is to study how obesity and a high fat diet might mediate abnormal reproduction in women, and pregnant women are not eligible for these studies. To avoid any possible exposure, participants will be included only if they are using a reliable non-hormonal method of contraception and they or their partner has been sterilized. They will only undergo frequent sampling studies in the early follicular phase of the menstrual cycle, and thus are extremely unlikely to be pregnant with a normal gestation if they have just had a menstrual period. As a further safeguard, we will verify the menstrual history with the participant before each frequent sampling study and perform a high-sensitivity urine pregnancy test (40 mIU/mlof hCG) prior to beginning any of the blood sampling sessions.

***Risks of frequent blood sampling:*** This procedure is commonly associated with bruising and bleeding at the site of the IV line. Rarely, anemia can result if amounts of blood withdrawn are excessive. Participants will participate in 6 hour frequent blood sampling sessions. During these sessions an IV will be placed in a vein in their arm and small amounts of blood (a little less than 1 teaspoon) will be drawn approximately every ten minutes (less than 1 cup total over 6 hours, or approximately 1.2 full cups of blood to be drawn in the total of two, 6-hour sessions).

A CBC will be taken at the end of the gonadotropin pulsatility study to assure that the participant has not become anemic. Screening and safety lab tests are likely to require another 20cc and safety labs at the end of the study will require a further 20cc. The total amount of blood over approximately 12 weeks is within permissible guidelines. As is our practice, we pre-screen all participants for an adequate hemoglobin level prior to entry (11gm/dl or greater), and supplement with iron (325mg daily for 3 months). VAMP tubing will be used when a nurse is available. Gravity tubing will be used if a nurse is unavailable and the lab technician must perform the frequent sampling for visits 1.1 and 6.1. By using gravity tubing, there will be an additional 18 milliliters of blood taken per 6-hour frequent sampling visit (1.1, and 6.1). There will still be less than 2/3 cup of blood taken per visit, as stated in the consent form.

*Infiltration:* There is also a risk of infiltration, or blood backing up in the IV line and leaking into the surrounding tissue, which can cause bruising and/or discomfort.

***Risk of DEXA:*** DEXA is a way of looking inside the body by using X-rays. X-rays are a type of radiation. Your natural environment has some radiation in it. A DEXA will give the participant about the same amount of radiation that they would get from the environment in 4 days.

***Risk of muscle oxygenation***: This is a non-invasive test with no significant risk or discomfort.

***Risk of Loss of Confidentiality/Privacy:*** There is a risk that people outside of the research team will see the participant’s research information. Through operating in accordance with university standards and in compliance with HIPAA, we will do all that we can to protect their information, but it cannot be guaranteed.

Data will be stored in OnCore, which limits access to PHI through usernames, passwords, and access levels. Data may also be stored in Excel on an encrypted drive that will be password protected with access restricted to study personnel only and institutional or federal officials as required by institutional regulations or law. The shared drive is backed up every evening. Specimens are stored in a locked refrigerator or freezer in the PI’s laboratory, which is in turn restricted in access to outside individuals based on access badging. Specimens and data are stored coded with an alphanumeric participant identification code. This code is not linked to any identifiable data about the participant. The file that links identifying information to study ID is double password protected in a separate folder and kept apart from primary research specimen data.

Data and Safety Monitoring Plan: This proposal involves substantial participant commitment and requires adequate recruitment of participants to be successful. Multiple medications and procedures will be administered to study participants. Lynda Barbour, MD and Theresa Powell, PhD, have agreed to serve as safety officers for this study. The safety officers will be provided with monthly recruitment updates and will be provided with any adverse event reports as they are submitted to the IRB. An initial meeting with the Safety Officer will be held before any subject enrollment in the study occurs in order for the Safety Officer and study team to establish a meeting schedule, review the study protocol, and study/participant termination guidelines. The Safety Officer will meet after the first 5 participants have been enrolled, and then after the enrollment of every 10 additional participants. They will assist the PI by making recommendations designed to improve recruitment or enhance the safety of the study for all participants. This is not a clinical trial and none of the information generated by the study will be used to promote drug marketing for any of the agents that are used.

***Potential Scientific Problems***

Aim 1:

Health-conscious women may not want to do a study that will involve high fat feeding, which is viewed as unhealthful. On the other hand, high fat food is generally quite tasty, the diet will be eucaloric and short term, which is exceedingly unlikely to have any harmful, long-term effects, and all food will be provided to participants for an entire month. Others have used diets as high as 45% fat in pregnant women with gestational diabetes with good outcomes, further evidence that a short term exposure to a diet high in fat will not cause long term adverse health consequences^9^. We have chosen to utilize rigorous methods to check for compliance. Variability at baseline in fat consumption will also be controlled by excluding women who are already consuming a HFD (defined as >40% energy from fat). It is most important that we avoid any weight loss in the NWW consuming a high fat diet, as this would have the potential to confound our results. Weight loss has been associated with increased urinary gonadotropin excretion and decreased serum gonadotropins^46^. Thus, while we maintain a eucaloric diet in Aim 1b, we will also weigh all of the participants weekly; assuring that they are consuming all of the food provided and measure fatty acid concentrations as a compliance marker.

Studies in lean men indicate unequivocal induction of increased hepatic insulin resistance after only 5 days of a eucaloric diet containing 65% calories from fat^10^, and non-statistically significant increases in both glucose and insulin have been observed after a 5-day high fat eucaloric diet in 12 women and men^11^. It is possible that women will be more resistant to a HFD intervention that is only 48% energy from fat. However, Bergougnian^1^, et al, have demonstrated clear-cut increases in FFAs after 72 hours on a eucaloric HFD diet identical to the one we plan to administer^8^. While we expect that the much longer exposure we propose (than what has been done in prior work) will likely be needed to achieve measurable changes in insulin sensitivity. We will not leave this to chance and have taken precautions described in the beginning of this aim to assure that we create a high fidelity model of hyperinsulinemia and elevated FFAs, which may require initial adjustment of our intervention (to include more simple carbohydrate or a brief period of hypercaloric consumption) before proceeding with the full experiment.

Aim 2:

We anticipate that a one-month exposure to an HFD will decrease insulin sensitivity, but outlined above in Aim 1, we will perform preliminary investigations to assure that the intervention is sufficient to cause detectable changes in gonadotropins. We will adjust our experimental design before we begin if it should it be necessary.

Aim 3:

Because this aim is relatively exploratory, it would be ideal to include as many analytes as possible. We shall seek to perform a more comprehensive cytokine analysis using a multiplex platform in addition to the ten-analyte panel described above to increase the number of potential relationships that will be detected.

***Data Analysis Plan***

Aim 1:

The primary outcome will be LH pulse amplitude as assessed in the non-intervention frequent sampling study and compared to the early follicular phase study under treatment (i.e., either insulin + FFAs for Aim 1A or HFD for Aim 1B). LH pulsatility will be analyzed using a modification of the original Santen and Bardin method, as previously described and utilized by the PI in numerous studies^1,6^. Up to 5 women will be assessed with frequent sampling prior to initiating the clamp portion of the study in order to confirm that diminished LH pulse amplitude is observed with the HFD. GnRH response will be compared between the non-intervention and intervention study as described above for gonadotropin pulsatility. We have used area under the curve methods to determine the LH response to exogenous GnRH and will utilize the same methodology as we have done in the past.^6^ FSH parameters will be compared between the non-intervention and intervention studies for both aims as described above for gonadotropin pulsatility. We will compare mean FSH, as pulsatility of FSH is less obvious than LH. We will compare changes in gonadotropin pulse frequency (for LH, and if we can detect distinct FSH pulses, we will compare FSH as well), mean LH and FSH and kinetics of LH, and if possible, FSH, before and after the intervention, as previously reported^7^.

For Aim 1a, our preliminary data indicate that a sample size of 10 women should be sufficient to demonstrate short-term changes in gonadotropin amplitude, as LH pulse amplitude correlates well with mean levels in this setting. Moreover, we will be using a paired analysis. For Aim 1b, a sample size of 20 is estimated to have 80% power to detect a 25% decrement in LH pulse amplitude on the HFD using a 2 sided paired t-test, assuming α=0.05, an untreated mean of 7IU/L, and a standard deviation of the difference of 1IU/ml. Urinary hormone profiles will be assessed for the entire cycle before and two cycles after initiation of the HFD using previously described menstrual cycle parameters suitable for urinary hormone determinations^42^. The presumptive day of ovulation, called the Day of Luteal Transition (DLT) will be determined for all cycles that demonstrate a Pdg increment consistent with ovulation. Follicular and luteal phase lengths will be calculated, as will integrated follicular, luteal and whole cycle LH, FSH, E1c and Pdg^4^. Whole-cycle parameters will be calculated and compared before and after HFD using a paired t-test for interval data, which can be reasonably fit by a linear model framework and in a linear regression model if adjustment for covariates is necessary. No statistical adjustment will be made for multiple comparisons of potentially covarying hormones. Hormones measured in daily urine will be modeled using linear mixed-effects regression to account for repeated measures nature of the data. Right-skewed variables will be analyzed on the log-scale, back-transformed and reported as geometric mean and 95% CI. We will also measure ovarian reserve markers: anti-Mullerian hormone (AMH; Ansh Laboratories), inhibin A and B (DSL, Webster, TX) before and after the intervention to determine whether they are altered by the intervention. All of these hormones have been previously measured by the PI^4,5^. Activity will be monitored monthly during the entire study, and weekly during the month of HFD in all participants. The baseline cycle of collection will be used as the participant’s benchmark and a deviation of >10% from baseline activity in any given week will alert the study staff to counsel the participant to maintain her usual level of activity.

Aim 2:

We do not have preliminary data on M or GRA in normal weight and obese reproductive aged women in our community with which to determine variation suitable for construction of sample size and power estimates. However, other, similar studies in normal weight, postmenopausal women in the Denver metro area have determined significant between group differences using a sample size of 12.^3,51^ We have therefore chosen a sample size of 15 to account for possible dropout, as paired analysis is essential for this aim. We also seek a slightly higher number in order to perform the association analyses described above.

Aim 3:

Exploratory Statistical Analysis: Testing the Mediation Hypothesis

Cytokines from the panel will be compared pre-and post-HFD using paired methodology. Cytokines that are significantly different after the HFD will be included in the mediation analysis. We will test that FFAs are correlated with change in the subset of selected cytokines. We will then test that change in the selected cytokines are correlated with drops in LH pulse amplitude. A linear regression model with change in LH pulse amplitude as the outcome will be estimated. Next a multivariable linear regression model with change in LH pulse amplitude as the outcome will be estimated, adjusted for change in cytokines. If multiple cytokines are identified for inclusion in this model, the correlation between change in cytokines will be assessed. If correlation between change in cytokines is greater than 0.3, a mixed model approach will be taken to control for cytokines and estimate the adjusted parameter for the effect of FFA on change in LH. Changes in inflammatory cytokines and adipokines will be associated with change in mean LH, LH pulse amplitude, estradiol (from the frequent sampling studies) and whole cycle integrated Pdg as above, using multiple linear regression modeling.

***Summarize Knowledge to be Gained***

Success of this project would represent a paradigm shift in the field, as we predict that adverse reproductive performance in obese women can be traced to one of its most proximal points—the pituitary gland—which then causes inadequate gonadal stimulation and disruption of the menstrual cycle. We seek to elucidate these processes by using a classical approach and inducing the defect in unaffected individuals. Demonstration that a eucaloric HFD (with or without other enhancements) can induce insulin resistance and/or elements of the reprometabolic syndrome in women will fill a glaring gap in knowledge that will point towards the potential impact of dietary intervention for the subfertility of obesity. These studies will provide mechanistic insights in its first two aims and unambiguous linkages of potential inflammatory mediators to the condition, all of which shall be mobilized with the ultimate goal of designing a feasible intervention that addresses as many elements of subfertility as possible.

Such an intervention could supplement weight loss and improve the ability of obese women not only to conceive, but to conceive a healthy pregnancy that is less likely to result in transgenerational transmission of obesity and dysmetabolism. Given the prevalence of obesity (about one third of the female population of the US) and the doubling of the likelihood of obesity in the offspring of obese mothers, application of the knowledge gained has the potential to have a multi-generational impact on the health of Americans. Moreover, the relative ineffectiveness of dietary interventions once obese women are already pregnancy speaks to the likelihood that the earliest events surrounding conception are affected by maternal obesity and that intervention must occur prior to conception.

**References**

1. Bergouignan A, Gozansky WS, Barry DW, et al. Increasing dietary fat elicits similar changes in fat oxidation and markers of muscle oxidative capacity in lean and obese humans. PloS one 2012;7:e30164.

2. Aubuchon M, Lieman H, Stein D, et al. Metformin does not improve the reproductive or metabolic profile in women with polycystic ovary syndrome (PCOS). Reproductive sciences 2009;16:938-46.

3. Van Pelt RE, Gozansky WS, Hickner RC, Schwartz RS, Kohrt WM. Acute modulation of adipose tissue lipolysis by intravenous estrogens. Obesity (Silver Spring) 2006;14:2163-72.

4. Jain A, Polotsky AJ, Rochester D, et al. Pulsatile luteinizing hormone amplitude and progesterone metabolite excretion are reduced in obese women. The Journal of clinical endocrinology and metabolism 2007;92:2468-73.

5. Roth LW, Allshouse AA, Bradshaw-Pierce EL, et al. Luteal phase dynamics of follicle-stimulating and luteinizing hormones in obese and normal weight women. Clinical endocrinology 2014;81:418-25.

6. Sumino H, Ichikawa S, Yoshida A, et al. Effects of hormone replacement therapy on weight, abdominal fat distribution, and lipid levels in Japanese postmenopausal women. International journal of obesity and related metabolic disorders : journal of the International Association for the Study of Obesity 2003;27:1044-51.

7. Tan SY, Batterham M, Tapsell L. Increased intake of dietary polyunsaturated fat does not promote whole body or preferential abdominal fat mass loss in overweight adults. Obesity facts 2011;4:352-7.

8. Howard BV, Van Horn L, Hsia J, et al. Low-fat dietary pattern and risk of cardiovascular disease: the Women's Health Initiative Randomized Controlled Dietary Modification Trial. JAMA : the journal of the American Medical Association 2006;295:655-66.

9. Hernandez TL, Van Pelt RE, Anderson MA, et al. A higher-complex carbohydrate diet in gestational diabetes mellitus achieves glucose targets and lowers postprandial lipids: a randomized crossover study. Diabetes care 2014;37:1254-62.

10. Boyle KE, Canham JP, Consitt LA, et al. A high-fat diet elicits differential responses in genes coordinating oxidative metabolism in skeletal muscle of lean and obese individuals. The Journal of clinical endocrinology and metabolism 2011;96:775-81.11. Lim CC, Mahmood T. Obesity in pregnancy. Best Practice & Research Clinical Obstetrics & Gynecology 2014.

12. Kort JD, Winget C, Kim SH, Lathi RB. A retrospective cohort study to evaluate the impact of meaningful weight loss on fertility outcomes in an overweight population with infertility. Fertility and sterility 2014;101:1400-3.

13. Tanvig M, Vinter CA, Jorgensen JS, et al. Effects of Lifestyle Intervention in Pregnancy and Anthropometrics at Birth on Offspring Metabolic Profile at 2.8 Years: Results From the Lifestyle in Pregnancy and Offspring (LiPO) Study. The Journal of clinical endocrinology and metabolism 2015;100:175-83.

14. Wahl SM, Hunt DA, Wakefield LM, et al. Transforming growth factor type beta induces monocyte chemotaxis and growth factor production. Proceedings of the National Academy of Sciences of the United States of America 1987;84:5788-92.

15. Hassan MA, Killick SR. Negative lifestyle is associated with a significant reduction in fecundity. Fertility and sterility 2004;81:384-92.

16. Polotsky AJ, Hailpern SM, Skurnick JH, Lo JC, Sternfeld B, Santoro N. Association of adolescent obesity and lifetime nulliparity--the Study of Women's Health Across the Nation (SWAN). Fertility and sterility 2010;93:2004-11.

17. Frisco ML, Weden M. Early Adult Obesity and U.S. Women's Lifetime Childbearing Experiences. Journal of marriage and the family 2013;75:920-32. 68

18. Guerrero-Bosagna CM, Skinner MK. Epigenetic transgenerational effects of endocrine disruptors on male reproduction. Seminars in reproductive medicine 2009;27:403-8.

19. Papachatzi E, Papadopoulos V, Dimitriou G, Paparrodopoulos S, Papadimitriou-Olivgeris M, Vantarakis A. Prepregnancy maternal obesity and fetal-perinatal death in a Mediterranean country. Journal of perinatal medicine 2014.

20. Fedorcsak P, Dale PO, Storeng R, et al. Impact of overweight and underweight on assisted reproduction treatment. Human reproduction 2004;19:2523-8.

21. Schliep KC, Mumford SL, Ahrens KA, et al. Effect of male and female body mass index on pregnancy and live birth success after in vitro fertilization. Fertility and sterility 2015;103:388-95.

22. Legge A, Bouzayen R, Hamilton L, Young D. The impact of maternal body mass index on in vitro fertilization outcomes. Journal of obstetrics and gynaecology Canada : JOGC = Journal d'obstetrique et gynecologie du Canada : JOGC 2014;36:613-9.

23. Vinturache A, Moledina N, McDonald S, Slater D, Tough S. Pre-pregnancy Body Mass Index (BMI) and delivery outcomes in a Canadian population. BMC pregnancy and childbirth 2014;14:422.

24. Luke B, Brown MB, Stern JE, et al. Female obesity adversely affects assisted reproductive technology (ART) pregnancy and live birth rates. Human reproduction 2011;26:245-52.

25. Stuebe AM, Landon MB, Lai Y, et al. Maternal BMI, glucose tolerance, and adverse pregnancy outcomes. American journal of obstetrics and gynecology 2012;207:62 e1-7.

26. Legro RS, Dodson WC, Gnatuk CL, et al. Effects of gastric bypass surgery on female reproductive function. The Journal of clinical endocrinology and metabolism 2012;97:4540-8.

27. Sherman BM, Korenman SG. Measurement of serum LH, FSH, estradiol and progesterone in disorders of the human menstrual cycle: the inadequate luteal phase. The Journal of clinical endocrinology and metabolism 1974;39:145-9.

28. Williams NI, Leidy HJ, Hill BR, Lieberman JL, Legro RS, De Souza MJ. Magnitude of daily energy deficit predicts frequency but not severity of menstrual disturbances associated with exercise and caloric restriction. American journal of physiology Endocrinology and metabolism 2015;308:E29- 39.

29. Xia L, Matera C, Ferin M, Wardlaw SL. Interleukin-1 stimulates the central release of corticotropin-releasing hormone in the primate. Neuroendocrinology 1996;63:79-84.

30. Roth LW, Bradshaw-Pierce EL, Allshouse AA, et al. Evidence of GnRH antagonist escape in obese women. The Journal of clinical endocrinology and metabolism 2014;99:E871-5.

31. Tortoriello DV, McMinn J, Chua SC. Dietary-induced obesity and hypothalamic infertility in female DBA/2J mice. Endocrinology 2004;145:1238-47.

32. Lichtenstein AH, Schwab US. Relationship of dietary fat to glucose metabolism. Atherosclerosis 2000;150:227-43.

33. Gupte AA, Minze LJ, Reyes M, et al. High-fat feeding-induced hyperinsulinemia increases cardiac glucose uptake and mitochondrial function despite peripheral insulin resistance. Endocrinology 2013;154:2650-62.

34. Institute NC. Usual Dietary Intakes. 2007.

35. Marklund M, Magnusdottir OK, Rosqvist F, et al. A dietary biomarker approach captures compliance and cardiometabolic effects of a healthy Nordic diet in individuals with metabolic syndrome. The Journal of nutrition 2014;144:1642-9.

36. Santaren ID, Watkins SM, Liese AD, et al. Serum pentadecanoic acid (15:0), a short-term marker of dairy food intake, is inversely associated with incident type 2 diabetes and its underlying disorders. The American journal of clinical nutrition 2014;100:1532-40.

37. Bradbury KE, Skeaff CM, Crowe FL, Green TJ, Hodson L. Serum fatty acid reference ranges: percentiles from a New Zealand national nutrition survey. Nutrients 2011;3:152-63.

38. Folch J, Lees M, Sloane Stanley GH. A simple method for the isolation and purification of total lipides from animal tissues. The Journal of biological chemistry 1957;226:497-509. 69

39. Reece MS, McGregor JA, Allen KG, Harris MA. Maternal and perinatal long-chain fatty acids: possible roles in preterm birth. American journal of obstetrics and gynecology 1997;176:907-14.

40. Santoro N, Crawford SL, Allsworth JE, et al. Assessing menstrual cycles with urinary hormone assays. American journal of physiology Endocrinology and metabolism 2003;284:E521-30.

42. Santoro N, Adel T, Skurnick JH. Decreased inhibin tone and increased activin A secretion characterize reproductive aging in women. Fertility and sterility 1999;71:658-62.

43. Santoro N, Lo Y, Moskaleva G, et al. Factors affecting reproductive hormones in HIV-infected, substance-using middle-aged women. Menopause 2007;14:859-65.

44. Beitins IZ, Shah A, O'Loughlin K, et al. The effects of fasting on serum and urinary gonadotropins in obese postmenopausal women. The Journal of clinical endocrinology and metabolism 1980;51:26-34.

45. DeFronzo RA, Tobin JD, Andres R. Glucose clamp technique: a method for quantifying insulin secretion and resistance. The American journal of physiology 1979;237:E214-23.
